# Phase I Clinical Trial on Pleural Mesothelioma Using Neoadjuvant Local Administration of Paclitaxel-Loaded Mesenchymal Stromal Cells (PACLIMES Trial): Study Rationale and Design

**DOI:** 10.3390/cancers16193391

**Published:** 2024-10-04

**Authors:** Giulia Maria Stella, Daniela Lisini, Paolo Pedrazzoli, Giulia Galli, Chandra Bortolotto, Giulio Melloni, Gioacchino D’Ambrosio, Catherine Klersy, Amelia Grosso, Francesca Paino, Stefano Tomaselli, Laura Saracino, Giulio Alessandri, Augusto Pessina, Elena Grignani, Vittorio Rosti, Angelo Guido Corsico, Patrizia Comoli, Francesco Agustoni

**Affiliations:** 1 Department of Internal Medicine and Medical Therapeutics, University of Pavia Medical School, 27100 Pavia, Italy; p.pedrazzoli@smatteo.pv.it (P.P.); g.galli@smatteo.pv.it (G.G.); angelo.corsico@unipv.it (A.G.C.); f.agustoni@smatteo.pv.it (F.A.); 2 Unit of Respiratory Diseases, Cardiothoracic and Vascular Department, IRCCS Policlinico San Matteo, 27100 Pavia, Italy; a.grosso@smatteo.pv.it (A.G.); s.tomaselli@smatteo.pv.it (S.T.); l.saracino@smatteo.pv.it (L.S.); 3 Cell Therapy Production Unit, Fondazione IRCCS Istituto Neurologico Carlo Besta, 20133 Milan, Italy; daniela.lisini@istituto-besta.it; 4 Medical Oncology Unit, Oncology and Hematology Department, Fondazione IRCCS Policlinico San Matteo, 27100 Pavia, Italy; 5 Diagnostic Imaging Unit, Department of Clinical, Surgical, Diagnostic, and Pediatric Sciences, University of Pavia, 27100 Pavia, Italy; c.bortolotto@smatteo.pv.it; 6 Radiology Unit-Diagnostic Imaging I, Department of Diagnostic Medicine, Fondazione IRCCS Policlinico San Matteo, 27100 Pavia, Italy; 7 Unit of Thoracic Surgery, Cardiothoracic and Vascular Department, IRCCS Policlinico San Matteo, 27100 Pavia, Italy; g.melloni@smatteo.pv.it; 8 Pathology Unit, Department of Diagnostical Services and Imaging, Fondazione IRCCS Policlinico San Matteo, 27100 Pavia, Italy; g.dambrosio@smatteo.pv.it; 9 Biostatistics and Clinical Trial Center, Fondazione Istituto di Ricovero e Cura a Carattere Scientifico Policlinico San Matteo, 27100 Pavia, Italy; c.klersy@smatteo.pv.it; 10 CRC StaMeTec, Department of Biomedical, Surgical and Dental Sciences, University of Milan, 20122 Milan, Italy; f.paino@smatteo.pv.it (F.P.); giulio.alessandri@istituto-besta.it (G.A.); a.pessina@unimi.it (A.P.); 11 Environmental Research Center, Istituti Clinici Scientifici Maugeri IRCCS, 27100 Pavia, Italy; e.grignani@icsmaugeri.it; 12 Phase 1 Clinical Trial Unit and Experimental Therapy, IRCCS Policlinico San Matteo, 27100 Pavia, Italy; v.rosti@smatteo.pv.it; 13 Cell Factory, Pediatric Hematology/Oncology, Fondazione IRCCS Policlinico San Matteo, 27100 Pavia, Italy; p.comoli@smatteo.pv.it

**Keywords:** pleural mesothelioma, cell therapy, local delivery, phase I study

## Abstract

**Simple Summary:**

The phase I monocentric PACLIMES trial evaluates the effects of local administration of an innovative tool composed of mesenchymal stromal cells loaded with paclitaxel directly injected in the pleural space in mesothelioma patients in a neoadjuvant setting. The trial explores the safety and toxicity of the drug product, but also an exploratory objective aimed at evaluating its antiproliferative capacity. The results of this study will validate a promising therapeutic option for an unmet clinical need such as pleural mesothelioma and open the way to an efficient management of malignant pleural effusion in case of advanced cancers from different sites of primary origin.

**Abstract:**

**Background and rationale.** Pleural mesothelioma (PM) is a rare and aggressive neoplasm that originates from the pleural mesothelium and whose onset is mainly linked to exposure to asbestos, which cannot be attacked with truly effective therapies with consequent poor prognosis. The rationale of this study is based on the use of mesenchymal stromal cells (MSCs) as a vehicle for chemotherapy drugs to be injected directly into the pathological site, such as the pleural cavity. **Study design.** The study involves the use of a conventional chemotherapeutic drug, Paclitaxel (PTX), which is widely used in the treatment of different types of solid tumors, including PM, although some limitations are related to pharmacokinetic aspects. The use of PTX-loaded MSCs to treat PM should provide several potential advantages over the systemically administered drug as reduced toxicity and increased concentration of active drug in the tumor-surrounding context. The PACLIMES trial explores the safety and toxicity of the local administration of Paclimes in chemonaive patients, candidates for pleurectomy. The secondary objective is to find the effective Paclimes dose for subsequent phase II studies and to observe and record the antitumor activity. **Future direction**. The experimental pre-clinical background and rationale are discussed as well.

## 1. Introduction

Pleural mesothelioma (PM) is a fatal asbestos-related malignancy originating from the mesothelial cells of the pleura [1]. Although asbestos has been banned in several countries worldwide for many years, PM incidence has also decreased very slowly in these countries due to the long latency of malignant transformation after exposure [2]. While a relevant number of genomic alterations are known to drive epithelial carcinogenesis, fewer data have been reported regarding PM onset. Therefore, at present, no actionable targets can be exploited to effectively treat PM and patients’ prognosis remains extremely poor [3]. Growing evidence suggests that asbestos-associated inflammatory reactions might induce the malignant transformation of mesothelial cells and that the unique pleural microenvironment is involved in inducing resistance to therapies, as usually reported in clinical settings [4]. Current therapeutic strategies against PM are defined according to a multimodal approach encompassing chemo- (immune)- treatments, surgery, and ionizing radiation. Numerous chemotherapeutic regimens have been tested in the past, but the results have generally been disappointing [5]. A platinum-based doublet containing a third-generation antifolate (pemetrexed or raltitraxed) is the first-line standard of care [6]. However, other chemotherapeutic agents—among which is paclitaxel (PTX)—have shown a certain level of activity [7]. Consequently, patients should be encouraged to enter experimental clinical studies. Nevertheless, in some instances, the best supportive care remains the most reliable choice [8]. Moreover, it is important to point out that PM proliferation is prevalent within the pleural space. The latter defines a kind of anatomical sanctuary, which makes it difficult to penetrate enough doses of drugs administered systemically. It is also conceivable that drugs should be diluted into the volume of free liquid that is very often associated with PM. On the other hand, this anatomical specificity supports a strong rationale for the use of drugs conveyed directly within the pleural cavity [9]. Adult mesenchymal stromal cells (MSCs) derived either from the bone marrow (BM) or from fat tissue have been suggested as suitable cell sources for cell-based therapies [10]. Because of their self-renewal, differentiation, and paracrine properties, MSCs possess therapeutic potential by themselves. However, MSCs can be also manipulated in vitro; they can be engineered to reach pathologic sites and deliver therapeutic molecules [11,12,13]. Notably, quite independently from homing capacity, the number of cells able to reach a malignant mass relies on the route by which they are administrated [14,15]. From this perspective, it should be underlined that after intravenous (i.v) injection, most of the cells remain trapped in draining organs and the number of therapeutically active MSCs is importantly reduced [16,17]. Otherwise, the main goal of cancer chemotherapy is to localize the drug effect in the tumor microenvironment to kill as many tumor cells as possible with the least collateral toxicity. Recently developed formulations of anticancer compounds, such as PTX bound to albumin nanoparticles, have shown several potential advantages over free-form drugs, such as (i) protecting drugs from degradation before they reach their target, (ii) increasing tumor drug absorption, (iii) allowing better control of the timing and distribution of drugs on tumor tissue, and (iv) the prevention of drug interaction with normal cells, reducing systemic toxicity overall. Similar results can be obtained by using MSCs for the delivery of anticancer agents. Drug-eluting cell therapy could have main value in those tumors, such as PM, that are orphans of an effective therapy. Moreover, chemotherapy mediated by MSCs does not prevent the conventional approach and can therefore be considered a valid option in adjuvant settings. In addition, the pleura space, due to its anatomical features, represents the ideal site for the local delivery of drug-loaded MSCs [18]. The aim of the PACLIMES phase I study against early-stage PM is thus to take advantage of locally administering a well-known chemotherapy agent, namely PTX, into the pleural space, in direct contact with transformed areas through a biocompatible vehicle represented by MSCs.

## 2. Pre-Clinical and Clinical Background

**Figure 1:**
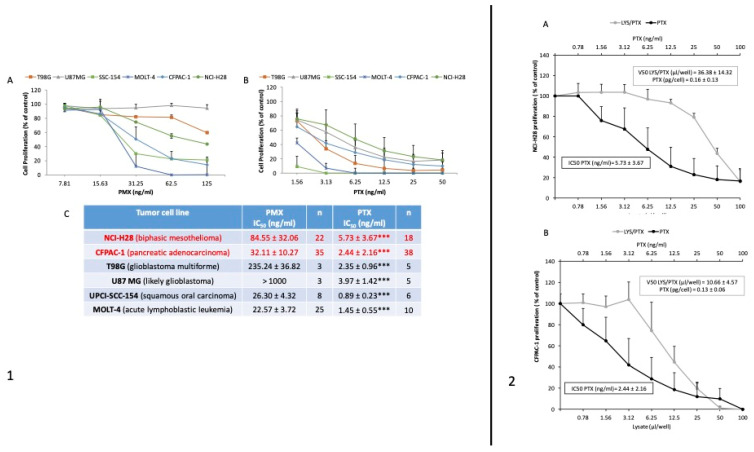
In vitro evidence of PTX and PTX-loaded MSCs on different cell lines, including NCI-H28 (PM). (**Panel 1**) Effects on tumor cell proliferation. The effects of increasing concentrations of PMX (**A**) and PTX (**B**) were evaluated with a 7-day MTT test. The effect was expressed as a percentage of the optical density measured in control cultures that did not receive PTX (100% proliferation). Box (**C**) reports the IC50 values, expressed as mean ± standard deviation (SD) of independent “n” experiments. Means were compared with a Student’s t test (*** *p* < 0.001). (**Panel 2**) MSCsPTX lysate tested on NCI-H28 (PM-) (**A**) and CFPAC-1 (pancreatic cancer) (**B**) cells. The antiproliferative effect of the lysate was evaluated in comparison to the effect of pure PTX. The dose-dependent inhibition was normalized to the antiproliferative activity of unloaded MSCs lysate, as a control condition. The graphs show IC50 values of PTX (ng/mL) and MSCsPTX lysates (µL/well) and the estimated amount of PTX released from a single loaded cell (pg/cell). Values are expressed as the mean ± SD of a series of three independent experiments.

Several preclinical data, already available in the literature, sustain the anti-oncogenic potential of MSC-carrying pharmacological agents, and their local delivery directly in contact with the transformed mass is considered a highly promising therapeutic approach [19,20,21,22]. Previous data from our group have demonstrated the anti-proliferative potential of MSCs upon in vitro exposure to very high doses of chemotherapeutic (e.g., PTX) [23,24,25]. We have reported in vivo models showing that both intra-tumor and systemic injection (i.v) of PTX-loaded MSCs can block malignant growth [26]. This property seems to be present not only in bone marrow-derived MSCs or cells obtained from adipose tissue but even in skin fibroblasts and monocytes [15,16]. In the last 20 years, it has been widely demonstrated that MSCs possess immunomodulatory functions, mainly via interactions with all immune cells (T-, B and Natural Killer cells, dendritic cells, monocytes, and others) and also through paracrine activity [27]. MSCs have been shown, both in vitro and in vivo, to inhibit naive and memory T-cell responses and to communicate with antigen-presenting cells by upregulating intercellular adhesion molecule-1 (ICAM-1) and vascular cell adhesion molecule-1 (VCAM-1), which are critical for T-cell activation and leukocyte recruitment to the inflammation site. MSCs also affect B-cells through cell-to-cell contact. MSCs also exert their immunomodulatory properties via paracrine mechanisms, secreting cytokines, growth factors, and chemokines, such as TGF- β1, TNF-α, PGE2, IFN-γ, and others, which combine to modulate the function of immune cells [28]. Based on these findings, MSCs have already been used worldwide in many clinical trials for the treatment of graft-versus-host-disease (GVHD), a serious complication that can arise after hematopoietic stem cell transplantation [29]. The results of clinical trials strongly demonstrated that MSCs are safe and well tolerated [30,31]. With respect to the choice of PTX, it has been mainly based on the following reasons: (i) it is a highly lipophilic drug so it can be well absorbed by cells; (ii) it is used for the treatment of many solid tumors and is able to inhibit the proliferation of endothelial cells (anti-angiogenic molecule); and (iii) unlike other chemotherapy drugs, it binds to the cytoskeleton and, by promoting the polymerization of microtubules, it induces the mitotic arrest of the cell; therefore, PTX is not a mutagenic (pro-neoplastic) compound. Moreover, to further support the study rationale, we investigated the activity of MSCs loaded with PMX and PTX. Drug release from MSCs-PMX and MSCs-PTX was tested by in vitro proliferation assays on a panel of tumor cell lines including NCI-H28 mesothelioma. The in vitro activity of PMX and PTX on the proliferation of different tumor lines was evaluated with MTT assays. Both PMX and PTX produced significant dose-dependent inhibition (*p* < 0.001), with greater activity exerted by PTX (the ratio between the IC50 values of the two drugs ranges from 13 to 100 ng/mL). The activity of PTX against NCI-H28 cells was approximately 13 times higher than that of PMX (Figure 1, panel 1). We also investigated if factors secreted by MSC could affect cell proliferation in vitro and we found a slight inhibitory effect (Figure 1, panel 2). By comparing the activity of pure PTX with that of MSCs-PTX, we estimated an average PTX per single cell equal to 0.15 pg. Based on this result, we can assume that with an in situ inoculation of 10^6^ cells per cm^3^ of tumor mass, we could reach a PTX concentration of approximately 150 ng/mL, which is 26 times higher than the IC50 determined in vitro [32,33]. The efficacy of inhibition of MSCs-PTX has then been verified in in vivo studies with MST0 211H biphasic human mesothelioma xenograft in nude mice by comparing the efficacy of free PTX inoculated intraperitoneally (i.p.) versus PTX loaded in human mesenchymal cells (MSCs-PTX). In consideration of the difficulties of using a pleural xenograft model, the study was performed with a subcutaneous transplant model of MSTO211H and subsequent loco-regional inoculation of MSCs-PTX or i.p. treatment of free drug. The therapeutic scheme involved four weekly treatments of 6–8 nude mice per experimental group with 2.5 × 10^8^ MSCs-PTX/kg (equivalent to 0.25 mg/kg of PTX = 0.05 mg/mouse) for in situ treatment and 20 mg/kg free PTX (clinical infusion form) (0.4 mg/mouse) for systemic treatment. Both the traditional i.p. treatment and treatment with MSCs-PTX in situ indicated a statistically significant reduction (*p* < 0.02) in neoplastic growth. The reduction was considered even after 14 days from the last treatment (day 21), indicating that the effect of controlling tumor growth by MSCs-PTX is comparable to that obtained by treating the animals with free PTX systemically. All preclinical in vivo data have shown that in situ treatment with MSCs-PTX has a therapeutic efficacy comparable to that observed with the treatment of free PTX i.p [23]. The therapeutic efficacy of MSCs-PTX is therefore comparable to systemic i.p treatment, with free PTX obtained with PTX dosages approximately 80 times lower. The result is also important in terms of a reduction in the cytotoxicity of PTX as indirectly indicated by the plasma concentration of PTX, which, in treatment with MSCs-PTX, appears 33.5 times lower than that achieved with i.p. treatment. Since PM is a tumor with a highly poor outcome even in the canine species, here, we reported a case we studied that concerned a canine mesothelioma treated with microfragmented adipose tissue loaded with PTX (MIFAT-PTX), already used for pharmacokinetic studies in mice [34]. A 24 kg dog suffering from pleural, pericardial, and peritoneal mesothelioma was treated with monthly intracavitary injections of MIFAT guided by ultrasound equivalent to 0.292 mg/kg. The patient was followed throughout the entire period of therapy with CT scans and radiographs and showed a lasting improvement in general conditions and excellent tolerability in the absence of toxicity or hypersensitivity. The clinical efficacy study in the dog allowed us to confirm and deepen the experimental pharmacokinetic data in the mouse. This study confirmed a low localization of the drug in the circulatory system and a propensity for diffusion into extravascular anatomical compartments. The behavior is compatible with the lipophilic structure of PTX and, presumably, is favored by the loco-regional route of administration and by the release of PTX previously incorporated from the biological scaffold of MSCs. The lower plasma concentration of PTX is closely related to lower systemic toxicity, without significant effects on granulocyte and platelet counts throughout the treatment. Of note is the presence of significant residual quantities of PTX found both in the pleura and in the pericardium one month after treatment, which confirms that loco-regional (intrathoracic) treatment translates into low levels of free drug circulating for a short period (8 h), but at a local concentration that is maintained at pharmacologically active levels [27,35]. Notably, our study investigates patients that could undergo pleural talcage after the experimental treatment according to national and international guidelines (e.g., National Comprehensive Cancer Network (NCCN), website at https://www.nccn.org/guidelines/category_1 (accessed on 1 September 2024); Associazione Italiana Oncologia Medica (AIOM), website at https://www.aiom.it/en/linee-guida-aiom/ (accessed on 1 September 2024)). This procedure is frequently used in the routine clinical settings in PM patients to obtain chemical pleurodesis; thus, the possible interaction between PTX and talc has been evaluated. The experimental in vitro results showed that about 85% of the PTX added to cell medium within talc is bound to the talc itself but that the aggregate particles feature significant antiproliferative activity (unpublished data from Pessina A et al.). Overall, although there is no direct correspondence with MSCs, the paper by Zaira et al. [27] demonstrates that even the entire adipose tissue—in which many MSCs are contained—functions as local drug delivery of PTX. In conclusion, in the present clinical trial, it should be noted that (i) the peculiar allogenic context significantly reduces the probability of a finalist MSC proliferation and (ii) the Paclimes tool is made with a dosage of PTX able to block MSC migration capacity. In other words, already published data and experimental pre-clinical findings allow us to conclude that MSCs in the Paclimes tool behave as inert vehicles of PTX while retaining the capacity to deliver it directly near the tumor mass.

Paclitaxel is approved for clinical use in several cancer types and acts as an antimicrotubule agent [36]. As above indicated, in 2005, the Food and Drug Administration (FDA) approved a new type of formulation called nanoparticle albumin-bound paclitaxel, or nab-paclitaxel, in which the drug is contained in tiny particles of protein. This improves drug delivery and reduces side effects [37]. In the clinical setting, PTX is a drug used in mesothelioma treatment, as an effective substitute for PMX. Some peritoneal mesothelioma patients have benefited from follow-up paclitaxel treatments after surgery [38,39]. The proposed approach guarantees the avoidance of toxic effects related to chemo agents when administered by the systemic route, which usually requests a reduction in calculated PTX therapeutic dose. PTX and PMX act according to different mechanisms: PMX is an antifolate that inhibits multiple folate-dependent enzymes whereas paclitaxel belongs to the class of microtubule interfering agents. The identification of markers predictive of response to chemotherapy (e.g., thymidylate synthase (TS) is a potential predictor of outcome after PMX; excision repair cross-complementation group 1 (ERCC1) as related to response to platinum) has been deeply evaluated in mesothelioma, although their predictive value in individual patients has not been fully validated [40,41,42]. Chemotherapy is the application of selective toxicity against those cells that are replicating at the highest rates. Thus, resistance to chemotherapy is often intrinsic to slow-growing tumors secondary to the acquisition of spontaneous mutations [43] and is related to the selection of specific resistant phenotypes [44]. Overall, many mechanisms of resistance to antineoplastic chemo agents are related to the activation or presence of a membrane pump that removes the drug from the cytoplasm shortly after it enters the cell through passive diffusion.

## 3. Trial Design

**Figure 2:**
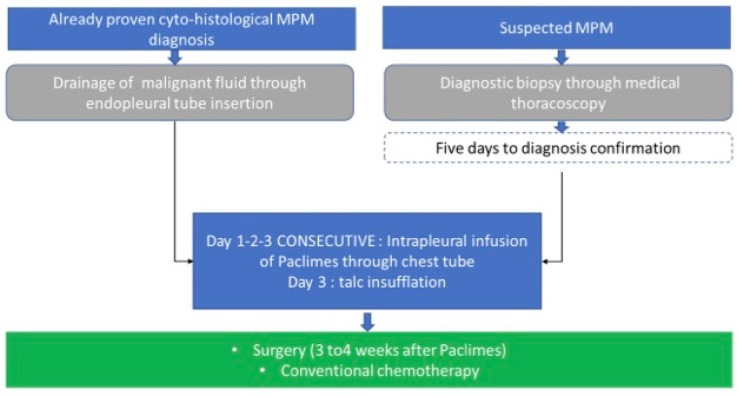
PACLIMES trial design. In the case of patients for whom a cyto-histological diagnosis of PM with concomitant pleural effusion will be available, endopleural drainage will be performed for fluid evacuation. Subsequently, we will proceed to the infusion through the drainage tube of 3/5 mL, containing a dose of PTX-loaded MV. The tube will be temporarily clamped, and the patient will be asked to change his decubitus. This procedure will be repeated in the following two days. Finally, at the end of the third infusion, we will proceed to talcum slurry. In the case of patients enrolled with a suspected PM diagnosis with associated pleural effusion, medical thoracoscopy will be performed and biopsies will be obtained. A drainage tube will be left in the cable until diagnosis confirmation (5 days). Then, the PTX-loaded MV will be infused as described above and followed by a powdery slurry. In any case, endopleural drainage will be removed two/three days after the powder and patients will be subjected to standard procedures.

**Figure 3:**
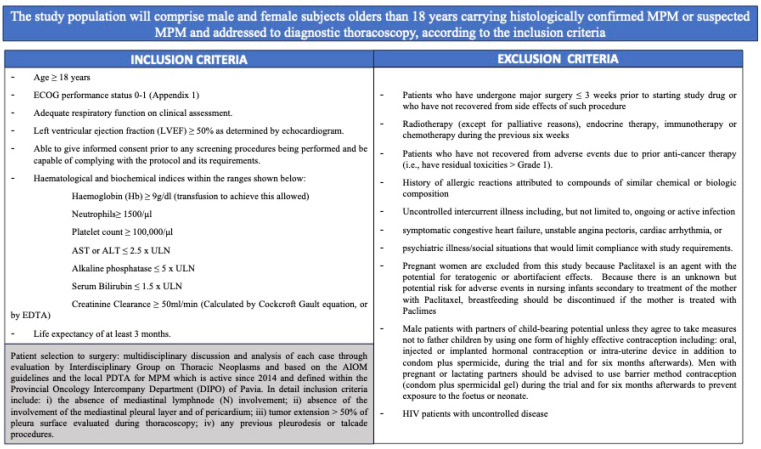
Inclusion and exclusion criteria for patients’ enrolment.

PACLIMES is a phase I open-label not-randomized monocentric trial. The study design is represented in Figure 2. Since Paclimes has never been tested in humans before, we have designed a phase I dose-escalation study to evaluate the maximum tolerated dose (MTD). The treatment duration is three days for each of the enrolled subjects. Inclusion and exclusion criteria are defined in Figure 3. Coherently to national and international guidelines on PM management, each case will be evaluated and followed by a dedicated multidisciplinary tumor board [8]. Then, each patient will continue the standard follow-up routinely planned for the disease. Notably, the patients will be treated with adjuvant chemotherapy according to the standard schedule (platinum-pemetrexed). During the course of the trial, a patient may withdraw treatment or consent at every step of the trial. The primary objective of the study is to assess the safety and toxicity profile and to evaluate the maximum tolerated dose of the local administration of Paclimes in patients with advanced chemo-naïve malignant PM. The secondary objective is to preliminarily assess the efficacy measured as relapse-free survival. To further validate the study hypothesis, which is limited by the difficulty of developing preclinical models that simulate the clinical setting of PM patients who are candidates for radical surgery, in addition to the primary objective of safety, the protocol aims to produce exploratory findings that, if safety is demonstrated, can be of help in the further development of this approach as a therapeutic option for the disease. To evaluate the degree of the induced tumor damage, a retrospective comparison with previous cases of which a complete pathological review can be carried out will be performed. Due to the difficulties in identifying and enrolling PM carrying early-stage disease, a prospective comparison with a randomized control group appears to be higher and more complex. We have identified a retrospective cohort of 10 PM cases from 2018 to 2021 who came to our observation and were diagnosed with PM, featuring clinical and TMN disease characteristics comparable to those of the population in the study and were similarly addressed to surgery in a neoadjuvant setting after multidisciplinary judgment and based on their good performance status and early phase tumor diagnosis. For each retrospective case, exhaustive surgical formalin-fixed paraffin-embedded (FF-PE) samples from pleurectomy will be analyzed, whereas, for each PM case enrolled in the trial, the baseline diagnostic specimens obtained through thoracoscopy and the matched surgical ones obtained after the experimental treatment will be analyzed. For both the retrospective arm and the study one, whole-slide digital scans will be acquired to accurately capture minute details at high resolution. This approach will allow a unique two-fold opportunity (i) to evaluate markers of potential predictive value in response to the local neoadjuvant treatment and (ii) to reduce the risk of an over-stated interpretation of any positive results in terms of safety of the study itself. To define the degree of tumor damage, the following markers will be analyzed and quantified. Morphology: (1) Presence of neoplastic cellular elements in apoptosis (defined by the following features: pyknotic nuclei, condensed cytoplasm, round cell fragments, staining agents do not penetrate the cell, the cell membrane is not permeable to staining agents, intact, compact organelles, ergastoplasmic dilation, intact cytoplasmic membrane, capsular and toroidal chromatin condensations at nuclear level) and mitotic arrest (mitotic spindle block); (2) presence of stromal reaction, such as fibrosis and edema; this will be assessed with respect to the degree of tumor cell populating the fibrosis. Immunohistochemistry: expression markers of cellular apoptosis (bcl-2). A baseline threshold will be defined based on the results on the control arm; a difference in expression of each marker higher than 0.5 of standard deviation will—arbitrarily and based on the literature data (e.g., [45,46])—be considered as significant. This will allow us to better establish what level should be required to support further efficacy studies. This pharmacological tool will be conveyed within the pleura via the endothoracic drainage tube placed at the end of the routine diagnostic thoracoscopy procedure and before the pleural talcage [47].

### 3.1. Route of Administration and Maximum Dosage Allowed

The drug product (DP), named Paclimes, obtained with the procedure setup by the methodology patented by Besta Institute (PCT/EP2011/059626), consists of MSCs isolated and expanded from subcutaneous lipo-aspirated adipose tissue (Ad-MSCs), loaded with a PTX drug cryopreserved in 0.9% NaCl Physiological Solution added with human albumin at the final concentration of 5% and DMSO at the final concentration of 10% and packaged in 2 mL cryovials at a concentration of 40–45 × 10^6^/vial, 2 mL/vial. Upon culturing under GMP conditions, Ad-MSCs are loaded with clinical-grade Paclitaxel and stored frozen until used. After thawing, the product will be administered to the patients bearing mesothelioma. The production of Paclimes will be performed by a large-scale methodology in a bioreactor (Quantum Cell Expansion System, Terumo BCT) by applying the described procedure [48]. In detail, the preparation of the drug product, i.e., MSCs loaded with paclitaxel (MSCs-PTX), follows the Good Manufacturing Practice regulation, as required by the Italian regulatory agencies, and occurs through different manufacturing steps, as described by Lisini D. et al. 2020 [48]. First, MSCs are isolated from adipose tissue lipoaspirates received by an Italian tissue bank, after obtaining patient informed consent and controls required. MSCs are isolated and expanded in flasks until reaching at least 20 × 10^6^ cells. Primary cultures were analyzed for morphology, number/viability, population doubling time, and flow cytometry (CD90, CD73, CD105, CD31, CD34, and CD45). MSCs’ large-scale expansion and loading with PTX are carried out in a synthetic hollow fiber bioreactor that works in a sterile closed-loop circuit for media and gas exchange. The PTX loading of MSCs occurs in 24 h, replacing the complete medium used for MSC expansion with a medium containing PTX. The drug products are cryopreserved until use. Batch release tests include the evaluation by compendial methods of endotoxin content (LAL test), sterility, *Mycoplasms* presence evaluation, and adventitious viruses as well as identity by flow cytometry, PTX content by HPLC, and potency via inhibition of the cell proliferation of mesothelioma cell lines.

DP aliquots of at least three different lots will be used for each treatment. The Paclimes compound will be instilled directly into the pleural space through a chest tube. The latter will be inserted before and independently from the experimental trial in order to proceed to talc slurry insufflation or after the thoracoscopic procedure. We aim at exploiting the tube as a route through which the Paclimes compound can be instilled. The treatment will start before talc insufflation in the case of palliative pleural poudrage, when the PM diagnosis is already known or five days after the diagnostic thoracoscopy. Our protocol is based on three administrations of 1.2 × 10^8^ Paclimes cells in a time of 72 h. Depending on the amount of drug carried by MSC-PTX (0.1–0.05 pg/cell), in situ, each treatment can provide an amount of 6 to 12 µg of the drug equivalent to a local-regional PTX administration of 18–36 µg of PTX. The total amount of time the patient will be receiving the drug is three days. The patient enters the phase I study before the first administration of Paclimes (day 1) and ends it before talc insufflation (day 3). No formal clinical studies have been conducted on the interaction of paclitaxel with other medicinal products. The trial is being carried out under a Clinical Trial Authorization. Following their participation in the trial, patients will be expected to return to a normal standard of care. Tumor assessment will be repeated as per the schedule of events given or more frequently, if clinically indicated. All lesions measured at baseline must be measured at subsequent disease assessments and recorded on the scan reports.

### 3.2. Statistical Methodology

**Figure 4:**
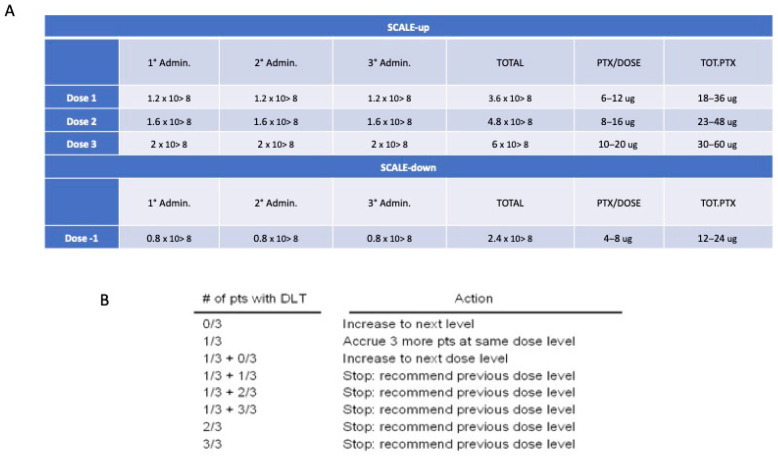
Dose finding approach. (**A**) Dose levels; (**B**) Number of patients according to the 3 + 3 design trial.

In a Phase I study designed to investigate the dose-limiting toxicity (DLT) and the maximum tolerated dose (MTD), a “3 + 3” cohort expansion design is used for the patients’ enrollment as described by Ivy et al. [49]. The therapeutic window for dose finding to better validate the relative safety of Paclimes is defined according to a variation of 33% from the starting dose (Figure 4). The dose is determined using dose-escalation strategies that target a toxicity rate of 33% or less. This target is reached by increasing the drug dose until the toxicity rate reaches 33% (i.e., 2 of 6 patients). According to the pragmatic “3 + 3” design, three patients are initially required. Treatment may then be escalated to the next higher dose in the absence of DLT; if a DLT is reported for one out of the three patients, the cohort is expanded to six patients to check the toxicity rate. If DLT is observed in >33% of patients, the current dose is unacceptably toxic and a dose level of -1 has to be applied. Once the potential therapeutic dose for a potentially subsequent phase II study is defined, a further cohort of 3 patients should/may be enrolled for dose confirmation (total 6 patients at the identified dose). The number of patients to be enrolled is 9 patients at minimum and 18 at maximum. The three-dose escalations scenario is detailed in Supplementary File S1.

## 4. Future Directions

Based on the above experimental pre-clinical and clinical evidence, we are therefore proposing to use MSCs loaded with PTX in the treatment of PM. The aim of the study is to take advantage of this disease-defined anatomical characteristic by introducing a well-known chemotherapy agent directly within the pleural space, in direct contact with PM areas. The drug will be delivered inside a biological vector, namely a stromal mesenchymal cell, capable in turn of modulating the peritumoral microenvironment to favor the cytostatic response. This approach, in the absence of additional risks associated with the administration of the experimental therapeutic tool, will assure several potential advantages over the free-form PTX such as the protection of the drug from degradation before reaching the target, the increase in drug concentration and uptake, a significant reduction in systemic toxicity, and the reduced amount of drug required to reach therapeutic doses. Overall, the results of this clinical study will open the way to an innovative and promising approach to managing malignant pleural effusions.

## Data Availability

The data that support the findings of this study are available on request from the corresponding author.

## Supplementary Materials

The following supporting information can be downloaded at https://www.mdpi.com/article/10.3390/cancers16193391/s1, File S1. Dose escalation scenario.
