# Supplementary material for: Phase I Clinical Trial on Pleural Mesothelioma Using Neoadjuvant Local Administration of Paclitaxel-Loaded Mesenchymal Stromal Cells (PACLIMES Trial): Study Rationale and Design
Source: Cancers (Basel). 2024 Oct 4;16(19):3391. doi: 10.3390/cancers16193391 (PMC11475395; doi:10.3390/cancers16193391)
Supplement: Supplementary file 1 [file cancers-16-03391-s001.zip › cancers-3180163-supplementary.pdf]

DOSE 1

1) Enroll 3 patients

- Scenario 1 - 0/3 patients show DLT: → toxicity rate 0 % (no patients reporting DLT), treatment can be escalated to the next higher dose level (DOSE 2).
- Scenario 2 - 1/3 patients show DLT: → toxicity rate ≤ 33%, stay at DOSE 1 and expand cohort (+3 patients) to verify toxicity rate.

2) Enroll 3 additional patients at the current dose for toxicity confirmation:

- Sub-Scenario 2.1 – 1/6 patients show DLT: toxicity rate 17% (no patients reporting DLT in the new cohort). Since toxicity rate is globally < 33% (1 patient reporting DLT in 6 patients enrolled) treatment can be escalated to the next higher dose level (DOSE 2).
- Sub-Scenario 2.2 – 2/6 patients show DLT: toxicity rate = 33% (1 patients reporting DLT in the new cohort). Since toxicity rate is globally equal to 33% (2 patients reporting DLT in 6 patients enrolled), the MTD is reached, therefore drug dose cannot be defined for a potentially subsequent phase II study. Dose escalation has to be stopped.
- Sub-Scenario 2.3 – 3/6 or 4/6 patients show DLT: toxicity rate > 33% (2 or 3 patients reporting DLT in the new cohort). Since toxicity rate is globally >33% (3 or4 patients reporting DLT in 6 patients enrolled), dose escalation has to be stopped. The current dose is unacceptably toxic. De-escalate to dose level -1
- Scenario 3 – 2/3 or 3/3 patients show DLT: toxicity rate > 33% (2 or 3 patients reporting DLT), dose escalation has to be stopped. The current dose is unacceptably toxic. De-escalate to dose level -1

DOSE 2

3) Enroll 3 patients:

- Scenario 1 - 0/3 patients show DLT: → toxicity rate 0 % (no patients reporting DLT), treatment can be escalated to the next higher dose level (DOSE 3).
- Scenario 2 - 1/3 patients show DLT: → toxicity rate ≤ 33%, stay at DOSE 2 and expand cohort (+3 patients) to verify toxicity rate.

4) Enroll 3 additional patients at the current dose for toxicity confirmation:

- Sub-Scenario 2.1 – 1/6 patients show DLT: toxicity rate 17% (no patients reporting DLT in the new cohort). Since toxicity rate is globally < 33% (1 patient reporting DLT in 6 patients enrolled) treatment can be escalated to the next higher dose level (DOSE 3).
- Sub-Scenario 2.2 – 2/6 patients show DLT: toxicity rate = 33% (1 patients reporting DLT in the new cohort). Since toxicity rate is globally equal to 33% (2 patients reporting DLT in 6 patients enrolled), the MTD is reached, dose escalation has to be stopped. The previous drug dose (DOSE 1) can be defined for a potentially subsequent phase II study. If only 3 patients were enrolled at DOSE 1 (scenario 1), enroll additional 3 patients at DOSE 1 for dose confirmation.
- Sub-Scenario 2.3 – 3/6 or 4/6 patients show DLT: toxicity rate > 33% (2 or 3 patients reporting DLT in the new cohort). Since toxicity rate is globally >33% (3 or 4 patients reporting DLT in 6 patients enrolled), dose escalation has to be stopped. The current dose is unacceptably toxic. The previous drug dose (DOSE 1) can be defined for a potentially subsequent phase II study. If only 3 patients were enrolled at DOSE 1 (scenario 1), enroll additional 3 patients at DOSE 1 for dose confirmation.
- Scenario 3 – 2/3 or 3/3 patients show DLT:
  toxicity rate > 33% (2 or 3 patients reporting DLT), dose escalation has to be stopped. The current dose is unacceptably toxic. The previous drug dose (DOSE 1) can be defined for a potentially subsequent phase II study. If only 3 patients were enrolled at DOSE 1 (scenario 1), enroll additional 3 patients at DOSE 1 for dose confirmation.

DOSE 3

5) Enroll 3 patients:

- Scenario 1 - 0/3 patients show DLT: → toxicity rate 0 % (no patients reporting DLT). The MDT cannot be defined. The current drug dose (DOSE 3) can be defined for a potentially subsequent phase II study. Enroll additional 3 patients at DOSE 3 for dose confirmation.
- Scenario 2 - 1/3 patients show DLT: → toxicity rate ≤ 33%, stay at DOSE 3 and expand cohort (+3 patients) to verify toxicity rate.

6) Enroll 3 additional patients at the current dose for toxicity confirmation:

- Sub-Scenario 2.1 – 1/6 patients show DLT: toxicity rate 17% no patients reporting DLT in the new cohort). Since toxicity rate is globally < 33% (1 patient reporting DLT in 6 patients enrolled). The MDT cannot be defined. The current drug dose (DOSE 3) can be defined for a potentially subsequent phase II study.
- Sub-Scenario 2.2 – 2/6 patients show DLT: toxicity rate = 33% (1 patients reporting DLT in the new cohort). Since toxicity rate is globally equal to 33% (2 patients reporting DLT in 6 patients enrolled), the MTD (maximally tolerated dose) is reached, dose escalation has to be stopped. The previous drug dose (DOSE 2) can be defined for a potentially subsequent phase II study. If only 3 patients were enrolled at DOSE 2 (scenario 1), enroll additional 3 patients at DOSE 2 for dose confirmation.
- Sub-Scenario 2.3 – 3/6 or 4/6 patients show DLT: toxicity rate > 33% (2 or 3 patients reporting DLT in the new cohort). Since toxicity rate is globally >33% (3 or 4 patients reporting DLT in 6 patients enrolled), dose escalation has to be stopped. The current dose is unacceptably toxic. The previous drug dose (DOSE 2) can be defined for a potentially subsequent phase II study. If only 3 patients were enrolled at DOSE 2 (scenario 1), enroll additional 3 patients at DOSE 2 for dose confirmation.
- Scenario 3 – 2/3 or 3/3 patients show DLT:
  toxicity rate > 33% (2 to 3 patients reporting DLT), dose escalation has to be stopped. The current dose is unacceptably toxic. The previous drug dose (DOSE 2) can be defined for a potentially subsequent phase II study. If only 3 patients were enrolled at DOSE 2 (scenario 1), enroll additional 3 patients at DOSE 2 for dose confirmation.
